# Lactose and Galactose Promote the Crystallization of Human Galectin-10

**DOI:** 10.3390/molecules28041979

**Published:** 2023-02-19

**Authors:** Yu-Fan Fu, Si-Cong Jiang, Zhong-Wei Zhang, Xin-Yue Yang, Zi-Lin Li, Jing Hu, Shu Yuan

**Affiliations:** 1 College of Resources, Sichuan Agricultural University, Chengdu 611130, China; 2 Haisco Pharmaceutical Group Comp. Ltd., Chengdu 611138, China; 3 Department of Cardiovascular Surgery, Xijing Hospital, Medical University of the Air Force, Xi’an 710032, China; 4 School of Medicine, Northwest University, Xi’an 710069, China

**Keywords:** crystallization, dimerization, electrostatic potential, galectin-10, lactose/galactose

## Abstract

Galectin-10 (Gal-10) forms Charcot–Leyden crystals (CLCs), which play a key role in the symptoms of asthma and allergies and some other diseases. Gal-10 has a carbohydrate-binding site; however, neither the Gal-10 dimer nor the CLCs can bind sugars. To investigate the monomer–dimer equilibrium of Gal-10, high-performance size-exclusion chromatography (SEC) was employed to separate serial dilutions of Gal-10 with and without carbohydrates. We found that both the dimerization and crystallization of Gal-10 were promoted by lactose/galactose binding. A peak position shift for the monomer was observed after treatment with either lactose or galactose, implying that the polarity of the monomer was reduced by lactose/galactose binding. Further experiments indicated that alkaline conditions of pH 8.8 mimicked the lactose/galactose-binding environment, and the time interval between monomers and dimers in the chromatogram decreased from 0.8 min to 0.4 min. Subsequently, the electrostatic potential of the Gal-10 monomers was computed. After lactose/galactose binding, the top side of the monomer shifted from negatively charged to electrically neutral, allowing it to interact with the carbohydrate-binding site of the opposing subunit during dimerization. Since lactose/galactose promotes the crystallization of Gal-10, our findings implied that dairy-free diets (free of lactose/galactose) might be beneficial to patients with CLC-related diseases.

## 1. Introduction

Charcot–Leyden crystals (CLCs) have been described as extracellular deposits of morphologically diverse crystals in inflamed tissues of patients [1,2], especially in eosinophils, macrophages, and basophils in the sputa of patients with bronchial asthma [3]. CLCs are considered hallmarks of eosinophil involvement in many diseases, such as allergic rhinitis [4], eosinophilic cystitis [5], atopic dermatitis [6], celiac disease [7], asthma [8], acute myeloid leukemia [9], colorectal cancer [10], mastocytoma [11], periapical lesion [12], and parasitic infections in the liver [13].

CLCs isolated from human eosinophils are composed of 1.2% carbohydrates, suggesting that they have lectin attributes [3]. The sequence of CLCs, with 142 amino-acid residues, is homologous to the family of galectins [14,15]. The crystal structure of CLCs [16] shows a highly similar structure to that found in other galectins [14]. Therefore, CLCs are also called galectin-10 (Gal-10) [17].

Galectins are a protein family with binding specificity to β-galactosides [15,18,19]. Galectins have been classified as proto-type, tandem-repeat-type, or chimeric-type structures [3]. Gal-10 falls into the proto-type category. Previous binding studies with simple sugars in the solid phase indicated that Gal-10 has weak but specific binding activity to lactose and N-acetyl-D-glucosamine [16,20]. Nevertheless, contradictory reports have suggested that this weak sugar-binding activity might be attributed to the binding of Gal-10 to the cross-linked agarose matrix [21]. Moreover, a crystal structure analysis of Gal-10 showed that it has no binding activity to β-galactosides but could bind mannose in a special pattern [22].

The Gal-10 dimer is formed by crystallographic symmetry. In the crystal structure, the putative sugar-binding site is on the dimer interface, and the Gal-10 dimers are packed in crystal forms [18,19,23]. Su et al. [18,19] found crystals of Gal-10 (crystallized with lactose) had water molecules and no lactose molecules in the sugar-binding site. In other words, neither the Gal-10 dimer nor the crystal can bind lactose. A detailed inspection of the Gal-10 dimer interface showed that Glu33 from the opposing subunit occupies a large area at the top of the Gal-10 sugar-binding site, which prevents sugars from interacting at the sugar-binding site. This may explain why previous studies were unsuccessful in co-crystallizing or adsorbing sugar molecules into Gal-10 crystals. Su et al. [18,19] produced several single-site variants, in which Glu33 was substituted with other amino acids. The structure of E33A, which has shorter side chains than the wild-type protein, recovered the ability to bind lactose [18,19]. A recent study suggested additional carbohydrate recognition sites at the peripheral surface of galectin-10 dimers in crystals [24]. However, this finding does not mean that lactose/galactose could bind to the interface between two galectin-10 monomers in a dimer.

Although Gal-10 cannot co-crystallize with carbohydrates, the effect of sugar molecules on the dimerization ratio or crystal size remains unknown. In this study, both the dimerization and crystallization of Gal-10 were promoted by lactose/galactose binding. An electrostatic potential shift indicated that the polarity of the monomer was reduced by the lactose/galactose binding, which may promote dimerization and crystallization.

## 2. Results

### 2.1. Gal-10 Is a Dimer at High Concentrations

**Figure 1:**
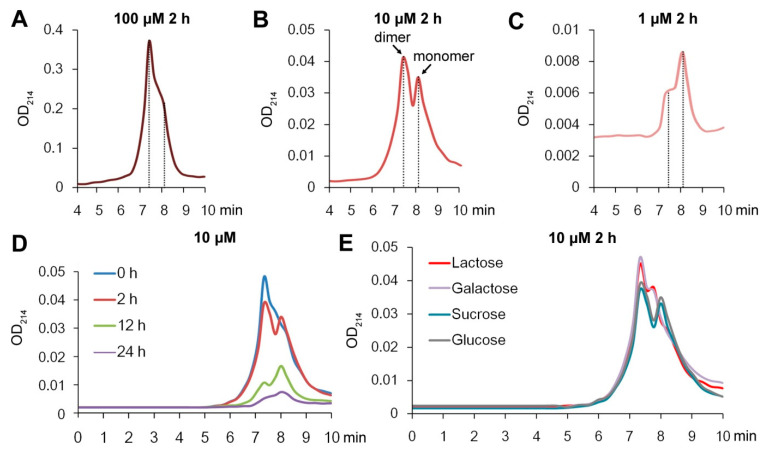
Concentration-dependent and time-dependent dimer formation of Gal-10 and the effects of carbohydrate binding. (**A**–**C**) Concentration-dependent dimerization of Gal-10. The protein was diluted to 100 μM, 10 μM, and 1 μM and incubated for 2 h. (**D**) Time-dependent dimer formation of Gal-10. The protein was diluted to 10 μM and incubated for 0 h, 2 h, 12 h, and 24 h. (**E**) Effects of sugars on Gal-10 monomer–dimer equilibrium. Gal-10 was diluted to 10 μM and incubated with 1 mM lactose, galactose, sucrose, or glucose for 2 h. The samples were analyzed by SEC HPLC using PBS–azide buffer (pH 7.4).

To investigate the monomer–dimer equilibrium of Gal-10, high-performance size-exclusion chromatography (SEC) was employed to separate serial dilutions of Gal-10 in PBS–azide buffer [25] with and without carbohydrates. A number of standard proteins ranging in size from 14 to 150 kDa were analyzed to standardize the size-exclusion properties of the SEC column (the column calibration line is shown in Figure S1). A mixing sample of 10 μM myoglobin and 10 μM Gal-10 verified the column performance (Figure S2). The diluted Gal-10 samples were incubated at 4 °C for 2 h to achieve equilibrium prior to analysis. Gal-10 proteins were recovered as two peaks at 7.4 min and 8.2 min, with a time interval of 0.8 min (Figure 1). The results were very similar to a previous report on human galectin-1 [25].

The monomer–dimer equilibrium was mainly dependent on the concentration of Gal-10. Most of the Gal-10 existed as dimers in the highest concentration solution of 100 μM (Figure 1A and Table S1). When the protein was diluted to 10 μM and incubated for 2 h, both monomers and dimers appeared (Figure 1B and Table S1), and at 1 μM, the monomers were the dominant form (Figure 1C and Table S1).

The rates of the formation of monomers from dimers were determined by diluting the dimers of the protein to 10 μM and detecting the conversion to monomers over time. Immediately after the samples were diluted (0 h), most of the Gal-10 existed as dimers (Figure 1D and Table S1). The equilibrium shifted gradually with time to the monomer form. After 2 h, a high concentration of monomers was detected, and after 24 h, the protein was mostly in the monomeric form (Figure 1D and Table S1). Interestingly, the amounts of both dimers and monomers gradually decreased, indicating increased crystallization with increasing incubation time. About 90% of the Gal-10 had formed crystals within 24 h (Figure 1D).

### 2.2. More Dimers Formed upon Lactose/Galactose Binding

To determine the effect of sugars on the monomer–dimer equilibrium for Gal-10, Gal-10 was diluted to the medium concentration of 10 μM and incubated with lactose, galactose, sucrose, or glucose at 4 °C for 2 h. The samples were analyzed by SEC HPLC using PBS–azide buffer either with or without sugars. The dimer formation of Gal-10 was not affected by sucrose or glucose (Figure 1E and Table S1). However, more dimers formed (monomers decreased) when either lactose or galactose was present (Figure 1E and Table S1). Interestingly, a peak position shift for the monomer (from 8.2 min to 7.8 min) was observed after either lactose or galactose treatment (Figure 1E). Besides the shape and size of the molecule, a change in electrical charge may also affect the retention time of SEC. The peak position shift may imply that the polarity of the monomer was reduced by lactose/galactose binding. The promotion of dimerization by lactose/galactose but not by sucrose/glucose was also confirmed with the native polyacrylamide gel electrophoresis (PAGE) method (Figure S3).

**Figure 2:**
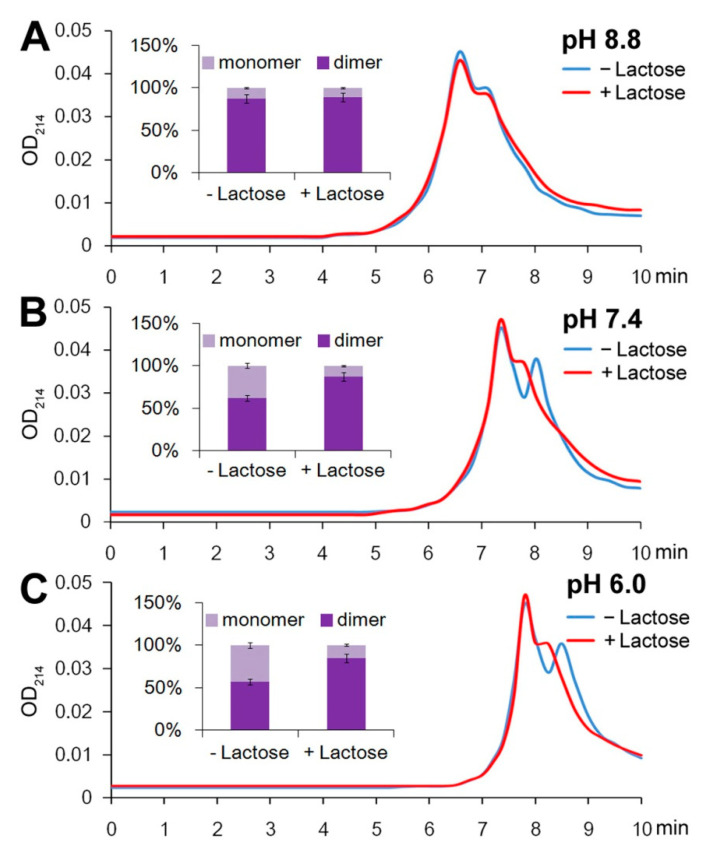
Effect of pH on Gal-10 monomer–dimer equilibrium. The protein was diluted to 10 μM in PBS–azide buffer at pH 8.8 (**A**), 7.4 (**B**), and 6.0 (**C**) with or without 1 mM lactose and incubated for 24 h. The samples were analyzed by SEC HPLC using PBS–azide buffer at pH 8.8 (**A**), 7.4 (**B**), and 6.0 (**C**). Quantitative data of Gal-10 monomers and dimers are shown on the left side of each panel. Bars represent the standard deviations of three independent replicates.

Further study indicated that alkaline conditions of pH 8.8 mimicked the lactose/galactose binding conditions, and the time interval between monomers and dimers in the chromatogram decreased from 0.8 min to 0.4 min (Figure 2A), indicating that the polarity of the monomer (but not the dimer) was reduced under the alkaline conditions. On the contrary, acidic conditions of pH 6.0 increased the retention time of both the monomer and dimer peaks, but the time interval between them was unchanged at pH 6.0 (Figure 2C). Given that the polarity of the monomer could be reduced at pH 8.8, we presumed that the Gal-10 monomer was negatively charged at pH ≤ 7.0, and upon lactose/galactose binding or alkaline treatment, its negative charge was partially neutralized.

Quantitative analysis suggested that in addition to the change in retention time, the dimerization of Gal-10 was also promoted by either lactose/galactose binding or alkaline treatment. When lactose was absent, the percentage of dimers increased from 57% at pH 6.0 to 62% at pH 7.4 or 87% at pH 8.8 (Figure 2A).

### 2.3. Lactose/Galactose Binding Promotes Gal-10 Crystallization

**Figure 3:**
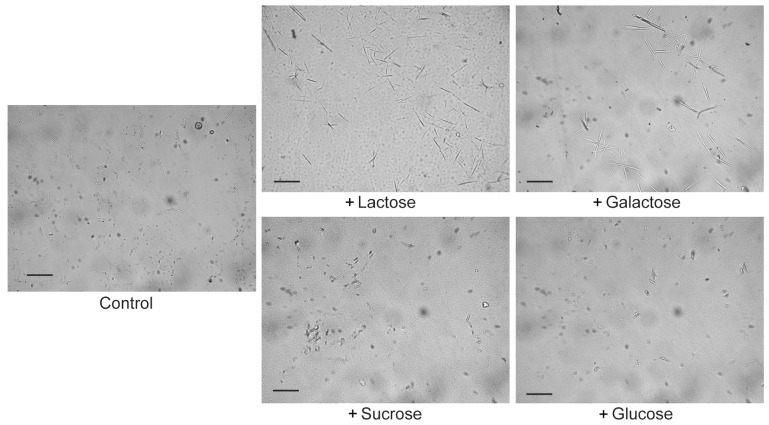
Effects of sugars on Gal-10 crystallization. Gal-10 was diluted to 10 μM in PBS–azide buffer with 0.01% Coomassie brilliant blue R-250 and incubated with or without 1 mM lactose, galactose, sucrose, or glucose for 24 h. The crystals were observed under a light microscope. Bar = 50 μM.

Gal-10 crystals resemble various crystal morphologies, such as spheres, spindles, and needle-like or rod-like shapes, as originally described by Charcot and Leyden [1,2]. The proteolytic removal of the N-terminal His-tag did not affect the heterogeneous shapes of the Charcot–Leyden crystals (Figure S4). In the control sample without carbohydrates, most crystals presented spheroidal or rod-like shapes with lengths less than 30 µm (Figure 3). Large spindle or needle-like crystals, with lengths greater than 30 µm, were only observed in 10 μM Gal-10 incubated with lactose or galactose for 24 h, but not in 10 μM Gal-10 incubated with sucrose or glucose for 24 h (Figure 3). This may have been because Gal-10 could not bind sucrose or glucose stably [18,19], although glucose might be absorbed on the peripheral surface of Gal-10 dimers in crystals [24].

**Figure 4:**
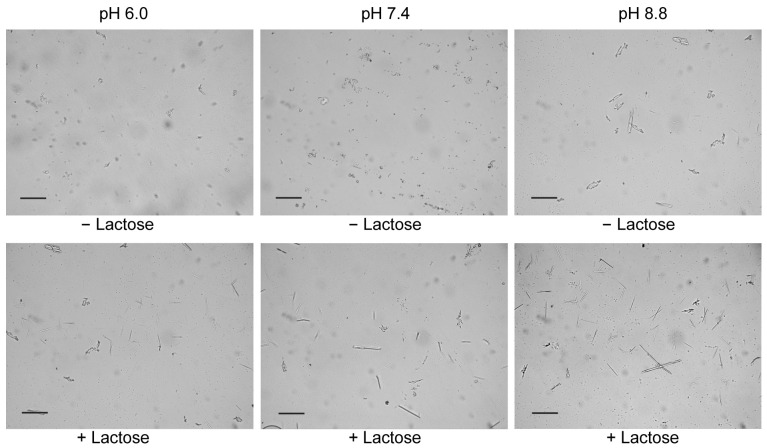
Effects of pH on Gal-10 crystallization. Gal-10 was diluted to 10 µM in PBS–azide buffer at pH 8.8, 7.4, and 6.0, with 0.01% Coomassie brilliant blue R-250 and incubated with or without 1 mM lactose for 24 h. The crystals were observed under a light microscope. Bar = 50 μM.

Besides carbohydrates, the pH value also affected the Gal-10 crystal morphology. When the pH of the incubation buffer was adjusted to 8.8, Gal-10 crystallization was promoted (crystals larger than 30 µm were observed), regardless of whether lactose was present (Figure 4). In contrast, at pH 6.0, Gal-10 crystallization was inhibited, especially in the absence of lactose (Figure 4). In general, crystallization was positively correlated with the dimerization ratio.

### 2.4. Surface Electrostatic Shift on the Gal-10 Monomer after Lactose Binding

Previous studies have demonstrated that all galectins have structure-conserved sugar-binding pockets [15,26]. The Trp72 residue in Gal-10 interacts with the pyranoside ring of galactose through the CH-π interaction. The His53 residue in Gal-10 at the base of the binding site forms a hydrogen bond with the O4 of galactose, and the Asn65 in Gal-10 that is proximal to His53 forms hydrogen bonds with the O3 and O4 of galactose. Gal-10 could co-crystallize with glycerol. Su et al. [18,19] found that Gal-10 had water molecules in the carbohydrate-binding sites, with His53 being the most crucial for binding, where glycerol would have been bound. The Trp72, His53, and Asn65 of Gal-10 are highly conserved residues in all galectins, and they might bind and stabilize galactose [15,18,19].

Su et al. reported that neither the Gal-10 dimer nor the crystal could bind lactose [18,19]. The authors explained that Glu33 from one Gal-10 monomer subunit may occupy a large area in the carbohydrate-binding site of the other subunit, such that the carbohydrate binding is hampered (or lactose is expelled from the Gal-10 monomer) during dimerization.

**Figure 5:**
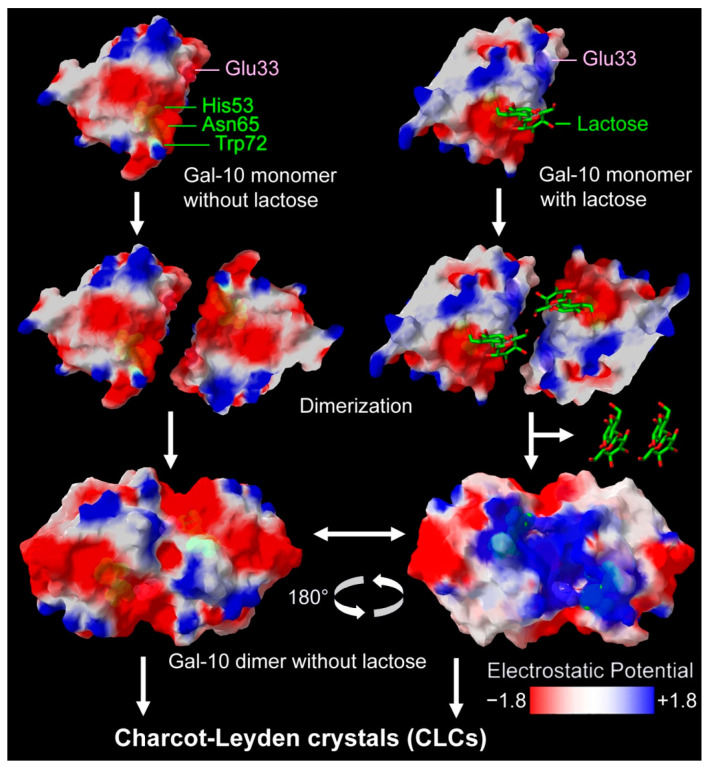
Shift in electrostatic potential may affect Gal-10 dimerization and crystallization. Given that Trp72, His53, and Asn65 bind with lactose (marked with green), the molecular surface and the electrostatic potential were re-computed by replacing Trp72, His53, and Asn65 with Ala to simulate the conditions of carbohydrate binding. Glu33 (marked with pale lavender) from one Gal-10 monomer subunit interacted with the carbohydrate-binding site of the opposing subunit when dimerizing. Then, lactose was expelled from the Gal-10 monomer after dimerization. The red-to-blue color gradient on the molecular surface indicates the electrostatic potential (red: −1.8; blue: 1.8).

The molecular surface and the electrostatic potential of the Gal-10 monomer were computed with Swiss-PdbViewer v4.1.0 software [27,28,29,30]. Since Trp72, His53, and Asn65 bind with lactose (galactose), the molecular surface and the electrostatic potential were re-computed by replacing Trp72, His53, and Asn65 with Ala to simulate the conditions of carbohydrate binding (the electric charge of these residues was neutralized by carbohydrate binding). As a result, a large part of the Gal-10 monomer surface was negatively charged, especially on the carbohydrate-binding site (Figure 5). Interestingly, besides Trp72, His53, and Asn65, the replacements altered the charge distribution of many other amino acid residues. For example, after lactose binding, the surface of the top of the protein shifted to electrically neutral where the Glu33 was distributed (Figure 5).

Before the replacements, both the carbohydrate-binding site and Glu33 were negatively charged, so they may have repelled each other and, therefore, prevented dimerization. On the contrary, the replacements reduced the negative charge on the area surrounding Glu33, which may have facilitated dimerization. However, whether lactose binding could induce a structural change that facilitates dimerization needs further investigation.

## 3. Discussion

There were at least five peaks in the 0 h profile for 10 μM Gal-10 (Figure 1D). The SEC-HPLC showed indistinguishable elution peaks. We thus verified the results with an alternative method of native PAGE (Figure S3). However, it was still difficult to obtain precise quantitative data. Future studies with HPLC-MALS (multi-angle light scattering) or HPLC-SAXS (small-angle X-ray scattering) [31,32] may more accurately determine the monomer/dimer dissociation–association properties.

The effect of lactose/galactose binding on the electrostatic potential distribution could be mimicked with alkaline conditions of pH 8.8. The Gal-10 monomer was negatively charged at pH ≤ 7.0, and upon lactose/galactose binding or alkaline treatment, its negative charge was partially neutralized. Thus, the lactose/galactose binding induced the electrostatic potential redistribution of the Gal-10 monomer. In particular, the topside of the monomer shifted from negatively charged to electrically neutral, allowing it to interact with the carbohydrate-binding site of the opposing subunit during dimerization.

A previous study presumed that carbohydrates bind to the peripheral surface of galectin-10 dimers in crystals, and that they may affect the stability of molecular packing in crystals, leading to the easy dissolution of CLCs and/or inhibiting the formation of CLCs [24]. However, the authors never proved this hypothesis. On the contrary, our results suggested that lactose/galactose promoted dimerization and subsequent crystallization by neutralizing the negative charges on the protein surface, especially in the area surrounding Glu33. Thus, lactose/galactose may not inhibit the formation of CLCs, but function as a catalyst for dimerization and crystallization.

Gal-10 plays a key role in the symptoms of asthma and allergic diseases [3,33]. Pathogen infections result in the excessive production of eosinophil in the respiratory system of the patient. A large number of eosinophils trigger asthma in the patient [34]. Blood leukocytes isolated from a patient of bronchial asthma with eosinophilic leukocytes contained about 75% eosinophils. Therefore, the blood lysate could easily form Gal-10 [35]. In some patients with both asthma and broncho-pulmonary infections, the Gal-10 levels were enhanced [36], implying that Gal-10 could be a biomarker of asthma [37]. Eosinophil-rich inflammation has been correlated with allergic diseases [38]. Gal-10 was detected in the tears of patients with vernal kerato-conjunctivitis (an allergic disease) [39]. Gal-10 was also present in nasal fluids from patients with seasonal allergic rhinitis during allergy season but was not present in samples collected before allergy season [40].

Although Charcot–Leyden crystal proteins are not a major determinant of human regulatory T-cell function (viability) [41], CLC is one of luminal-captured biomarkers of inflammation and eosinophilic esophagitis [42], and Gal-10 expression in the nasal polyps of patients with chronic rhinosinusitis was correlated with the severity of the disease according to Clinical-Cytological Grading [43]. Logistic regression analysis showed that crystalline CLCs in nasal tissues are predictive of nasal polyp recurrence. The occurrence of crystalline CLCs at a rate of more than one per high-power field could predict postoperative polyp recurrence with 84.80% sensitivity and 98.70% specificity [44]. At the intracellular level, CLCs are mostly stored in the peripheral cytoplasm of human eosinophils, being accumulated within an area about 250 nm wide underneath the plasma membrane but not within specific (secretory) granules. The high-resolution analysis of single cells revealed that CLCs interact with the plasma membrane, with immunoreactive microdomains of high CLC density being found in about 60% of the membrane area [45].

Exposure to ambient air pollutants may contribute to the pathogenesis of chronic rhinosinusitis and has been linked to both higher tissue inflammation and the presence of eosinophilic aggregates and CLCs in chronic rhinosinusitis patients [46]. Moreover, high levels of CLCs, chemokine (C-C motif) ligand 17, cystatin SN, interleukin (IL)-5, and macrophage inflammatory protein-1β in nasal secretions have been found to be associated with poor prognosis in chronic rhinosinusitis patients under surgical and conventional medical treatments [47].

Chronic rhinosinusitis with or without nasal polyps displays variable degrees of eosinophilic and neutrophilic inflammation, with a profound neutrophilic infiltration and activation in type 2 inflammation, associated with eosinophil extracellular trap cell death and CLC accumulation [48]. Macrophages, eosinophils, neutrophils, and non-inflammatory lung cells of acid sphingomyelinase-deficient mouse lungs exhibited the apparent accumulation of chitinase-like proteins, which formed large eosinophilic polygonal Charcot–Leyden-like crystals, indicating a connection between crystal-associated lung inflammation and alterations in macrophage function [49]. Besides the inflammation-related mechanisms, Gal-10 may also regulate the dynamic palmitoylation cycle, a post-translational modification that affects membrane localization, vesicular secretion, and transport, especially on the eosinophil’s plasma membrane [50]. CLCs endogenously express lysophospholipase activities, releasing free palmitate from substrate lysopalmitoylphosphatidylcholine. Palmitoylation then plays a regulatory role in the subplasmalemmal space of eosinophils, where the terminal vesicular secretory processes of piecemeal degranulation govern the secretory response of eosinophils [50]. Furthermore, CLCs interact with both human eosinophil granule cationic ribonucleases (RNases) and murine eosinophil-associated RNases. The interaction was found to be independent of glycosylation and not inhibitory toward endoRNase activity [51]. Thus, CLCs may function as carriers for the sequestration and vesicular transport of the potent eosinophil granule cationic RNases during both degranulation and differentiation, facilitating their intracellular packaging and extracellular function in allergic inflammation [51].

Lactose/galactose might aggravate asthma or allergies by inducing the production of Gal-10. A previous report indicated that dairy-free diets were associated with significant reductions in self-reported levels of nasopharyngeal secretions in people who formerly complained of persistent nasopharyngeal mucus hyper-secretion [52]. Another report found that low-fat yogurt intake was directly related to enhanced risks of both child asthma and allergic rhinitis [53]. However, without in vitro and/or in vivo experiments to correlate Gal-10 crystallization to dairy-free diets, we cannot simply recommend dairy-free diets for patients with bronchial asthma, seasonal allergic rhinitis, or other CLC-related diseases. More experimental and clinical investigations are needed.

Alternatively, drugs that restrict CLC accumulation may also be considered. For example, a Gal-10-specific antibody blocked CLC autocrystallization and relieved the inflammation in a humanized mouse model of asthma [23]. In another instance, Mepolizumab, a humanized antibody targeting interleukin-5, significantly decreased serum galectin-10 levels and therefore reduced the number of serum eosinophils and the frequency of severe asthma [54].

## 4. Materials and Methods

### 4.1. Human CLC Protein

Recombinant human Charcot–Leyden crystal (CLC) protein with a N-terminal His tag expressed in Escherichia coli was purchased from Cloud-Clone Corp. (Katy, TX, USA). The protein was purified with a Ni-nitrilotriacetic acid resin.

Recombinant His-tagged Gal-10 at 1 mg/mL was digested with TEV protease at room temperature for 12 h using a TEV protease: Gal-10 ratio of 1:100 (w:w) [23]. Then, Gal-10 proteins without His-tag were diluted to 10 μM for crystallization.

### 4.2. Separation of the Monomers and Dimers of Gal-10 via Size-Exclusion Chromatography

The separation of the monomers and dimers of Gal-10 was performed by size-exclusion HPLC (high-performance liquid chromatography) with a Spherogel-TSK-2000SW SEC column (7.5 mm × 60 cm, Beckman, Fullerton, CA, USA). The column was equilibrated with PBS–azide buffer (6.7 mM KH_2_PO_4_, 150 mM NaCl, 14 mM β-mercaptoethanol, 0.02% NaN_3_, pH 7.4) [25], and separation was performed isocratically for 20 min with a Beckman System Gold-126 HPLC (Beckman Coulter, Inc., Fullerton, CA, USA). The injection volume was 20 μL. The column flow rate was 1 mL/min. The absorbance of the fractions was monitored at 214 nm [25]. The column was calibrated with molecular mass markers of bovine γ-globulin (158 kDa), bovine serum albumin (67 kDa), chicken ovalbumin (44 kDa), equine myoglobin (25 kDa), chymotrypsinogen (17 kDa), myoglobin (16.7 kDa), and ribonuclease A (13.7 kDa).

### 4.3. Reversibility of Monomer–Dimer Equilibrium

Gal-10 was diluted to 100, 10, and 1 μM in PBS–azide buffer and incubated at 4 °C for 2 h or 24 h. To investigate the binding with carbohydrates, 10 μM Gal-10 was incubated with 1 mM lactose, galactose, sucrose, or glucose at 4 °C for 2 h. Then, the monomers and dimers were separated by SEC HPLC [25]. A number of standard proteins ranging in size from 14 to 150 kDa were analyzed to standardize the size-exclusion properties of the SEC column (the column calibration line is shown in Figure S1). Based on the elution positions of these molecular mass standards, it could be estimated that the first eluted peak of Gal-10 represented a 32 kDa protein (dimer), and the later peak represented a 16 kDa protein (monomer) [25]. To study the effect of pH on the monomer–dimer equilibrium, the pH of the PBS–azide buffer was adjusted to 6.0 or 8.8.

The monomer–dimer equilibrium was also determined by native polyacrylamide gel electrophoresis (PAGE) [55]. Gal-10 was diluted to 10 μM and incubated with or without 1 mM lactose, galactose, sucrose, or glucose for 2 h. After the native PAGE, the gel was stained with Coomassie Blue R-250.

### 4.4. Microscope Observation of Gal-10 Crystals

For co-crystallization with carbohydrates, 10 μM Gal-10 was incubated with 1 mM lactose, galactose, sucrose, or glucose in PBS–azide buffer with 0.01% Coomassie brilliant blue R-250 (to visualize the crystals) at 4 °C for 24 h. The formation of crystals was evaluated by observation under a light stereo-zoom microscope (Leica Microsystems M165C, Deerfield, IL, USA) equipped with a Leica IC80 HD camera [23,26]. To study the effect of pH on Gal-10 crystallization, the pH of PBS–azide buffer was adjusted to 6.0 or 8.8.

### 4.5. Homology Modeling of Gal-10

All full-length protein sequences were downloaded from the National Center of Biotechnology Information (NCBI; https://www.ncbi.nlm.nih.gov/) (accessed on 1 August 2021). The sequence of human Gal-10 protein (NP_001819.2) was analyzed by homology models, which were constructed in the SWISS-MODEL workspace (http://swissmodel.expasy.org/workspace/) (accessed on 1 August 2021) [27,28,29,30]. The optimal template was 5xrg.1.A, with a sequence identity of 97.89%. Both monomer and dimer templates were used for homology modeling.

The molecular surface and the electrostatic potential were computed with Swiss-PdbViewer v4.1.0 software [27,28,29,30] with the Poisson–Boltzmann equation [56,57]. To reveal all amino acids on the protein surface, the transparency of the surface was set to 30%.

Since Trp72, His53, and Asn65 bind with lactose and galactose [15,18,19], the molecular surface and the electrostatic potential were re-computed by replacing Trp72, His53, and Asn65 with Ala to simulate the conditions of carbohydrate binding (the electric charge of these residues would be neutralized by the carbohydrate binding).

### 4.6. Statistical Analysis

For all experiments, three independent replicates were performed. The data were statistically analyzed using two-way ANOVA with SPSS 22.0 software (IBM Comp., Chicago, IL, USA). Duncan’s multiple range test was performed to compare the means. The data were considered to be statistically significant at *p* < 0.05.

## Data Availability

Data are contained within the article.

## Supplementary Materials

The following supporting information can be downloaded at: https://www.mdpi.com/article/10.3390/molecules28041979/s1, Figure S1: The column calibration line of SEC, Figure S2: SEC HPLC of Gal-10 and myoglobin, Figure S3: Native PAGE of Gal-10 monomers and dimers, Figure S4: Effect of galactose on crystallization of Gal-10 proteins without His-tag, Table S1: Quantitative data (percentages) of Gal-10 monomers and dimers as shown in Figure 1.
