# Supplementary material for: Lactose and Galactose Promote the Crystallization of Human Galectin-10
Source: Molecules. 2023 Feb 19;28(4):1979. doi: 10.3390/molecules28041979 (PMC9966682; doi:10.3390/molecules28041979)
Supplement: Supplementary file 1 [file molecules-28-01979-s001.zip › molecules-2154981-supplementary.pdf]

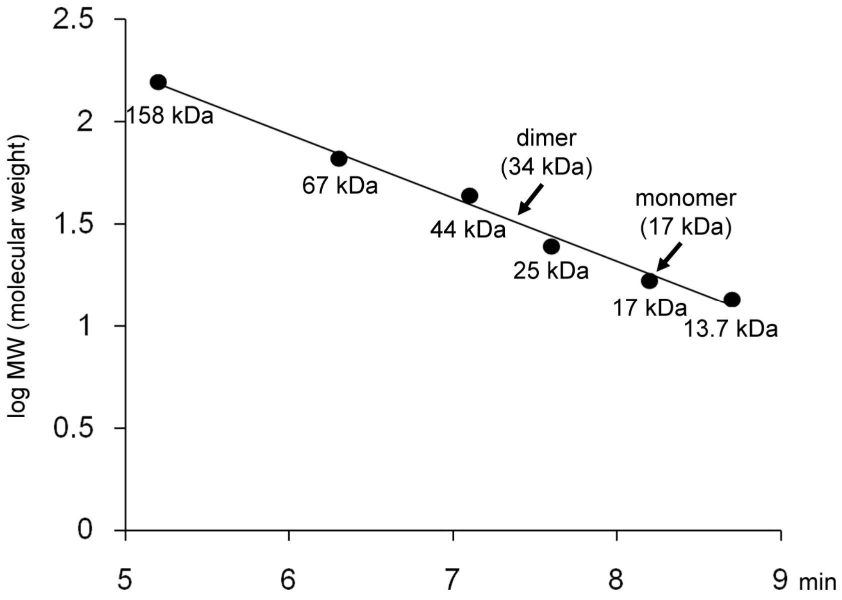

Figure S1. The column calibration line of SEC. Separation of the monomers (17 kDa) and dimers (34 kDa) of Gal-10 was performed by size-exclusion HPLC with a Spherogel-TSK-2000SW SEC column. The column was calibrated with molecular mass markers as indicated in the figure.

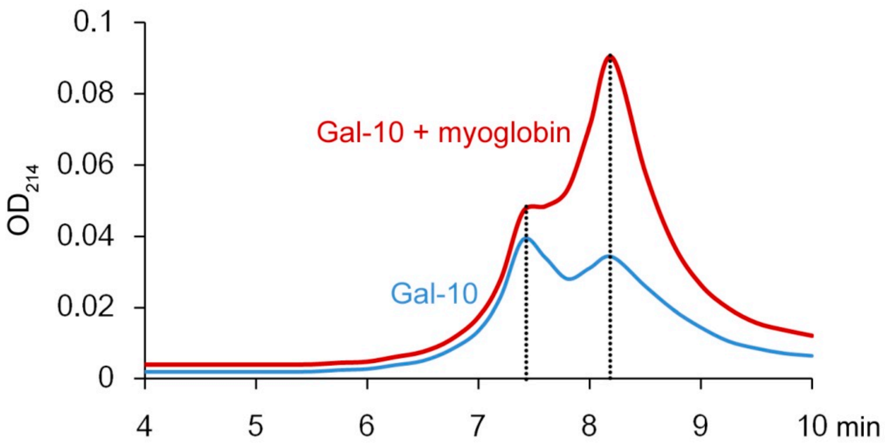

Figure S2. SEC HPLC of Gal-10 and myoglobin. Gal-10 was diluted to 10 μM and incubated for 2 h. Then the Gal-10 monomers (17 kDa) and dimers (34 kDa) with or without 10 μM myoglobin (16.7 kDa) were subjected to SEC HPLC with the PBS–azide buffer (pH 7.4).

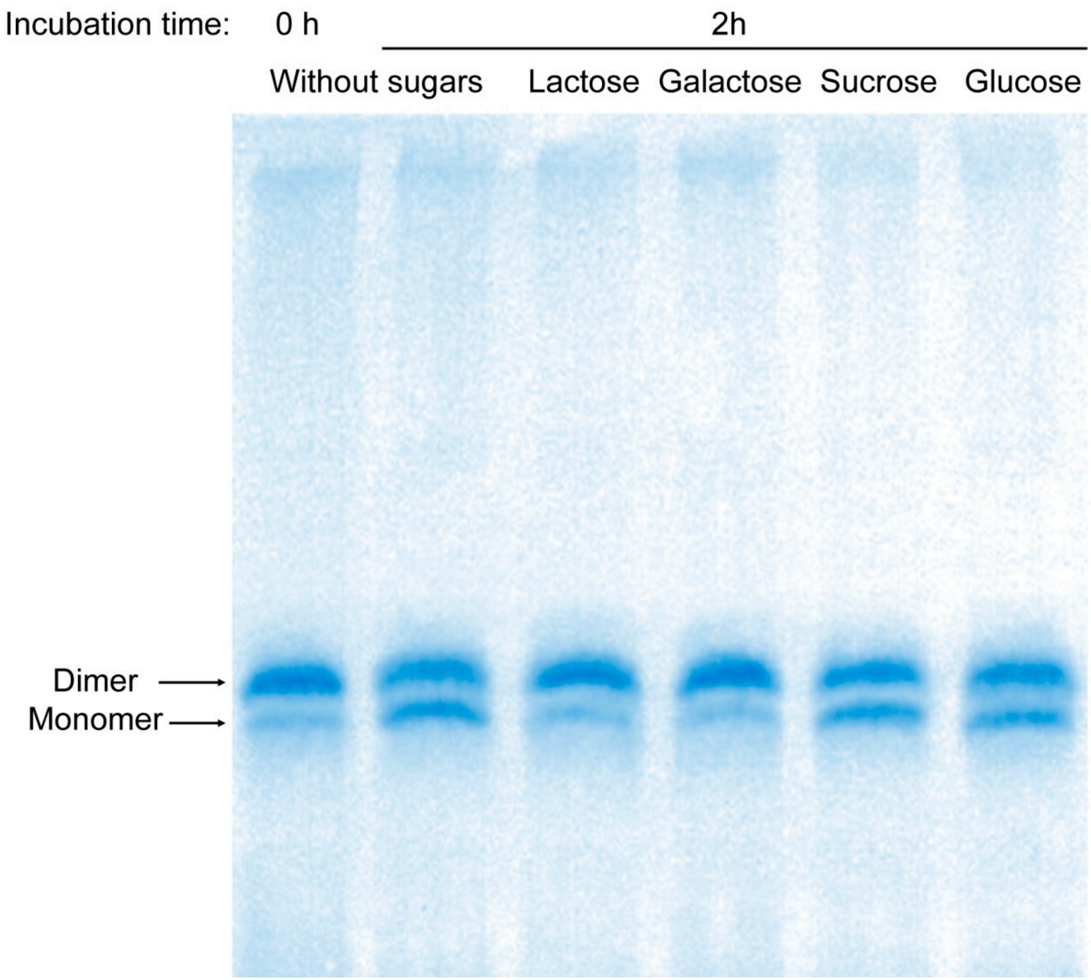

Figure S3. Native PAGE of Gal-10 monomers and dimers. Gal-10 was diluted to 10 µM and incubated with or without 1 mM lactose, galactose, sucrose, or glucose for 2 h. For analyzing the monomer–dimer equilibrium of Gal-10, the gel was stained with Coomassie Blue R-250.

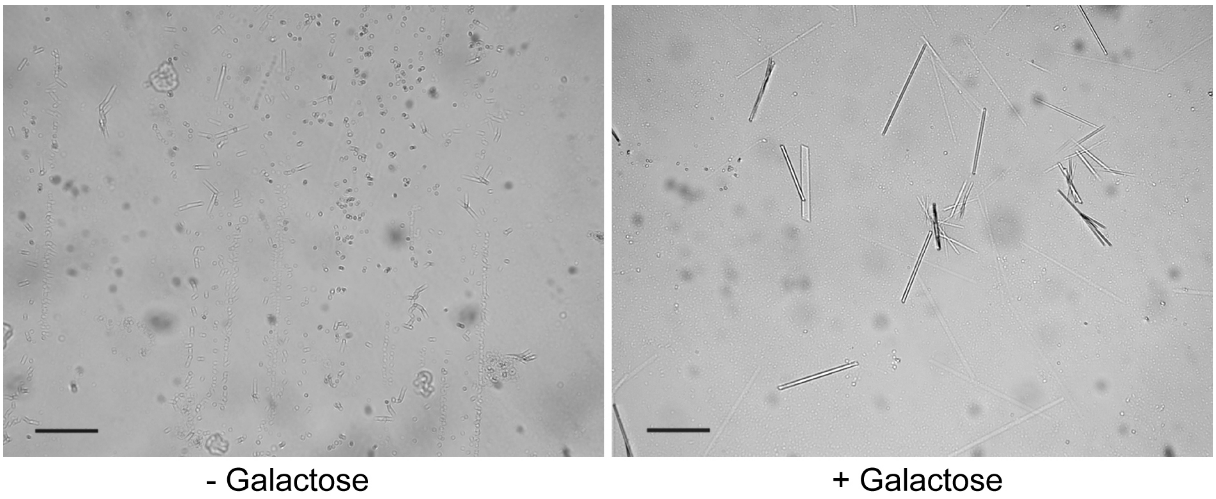

Figure S4. Effect of galactose on crystallization of Gal-10 proteins without His-tag. Recombinant His-tagged Gal-10 was digested with TEV protease to remove the tag. Then Gal-10 without His-tag was diluted to 10 μM in PBS–azide buffer with 0.01% Coomassie brilliant blue R-250 and incubated with or without 1 mM galactose for 24 h. The crystals were observed under a light microscope. Bar = 50 μM.

Table S1. Quantitative data (percentages) of Gal-10 monomers and dimers as shown in Figure 1.

Treatment	dimer	monomer
100 µM Gal-10 for 2 h	89%±2%	11%±2%
10 µM Gal-10 for 2 h	62%±4%	38%±4%
1 µM Gal-10 for 2 h	18%±3%	82%±3%
10 µM Gal-10 for 0 h	95%±1%	5%±1%
10 µM Gal-10 for 12 h	28%±3%	72%±3%
10 µM Gal-10 for 24 h	10%±2%	90%±2%
10 µM Gal-10 with 1 mM lactose for 2 h	87%±2%	13%±2%
10 µM Gal-10 with 1 mM galactose for 2 h	88%±2%	12%±2%
10 µM Gal-10 with 1 mM sucrose for 2 h	63%±4%	37%±4%
10 µM Gal-10 with 1 mM glucose for 2 h	63%±4%	37%±4%

Mean values with the standard deviations of three independent replicates are shown.
